# The use of patient-reported measures in epilepsy care: the Calgary Comprehensive Epilepsy Program experience

**DOI:** 10.1186/s41687-021-00356-4

**Published:** 2021-10-12

**Authors:** Guillermo Delgado-García, Samuel Wiebe, Colin B. Josephson

**Affiliations:** 1 Department of Clinical Neurosciences, Cumming School of Medicine, Foothills Medical Centre, University of Calgary, 1403 - 29 St NW, Calgary, AB Canada; 2 Centro de Investigación y Desarrollo en Ciencias de la Salud (CIDICS), Universidad Autónoma de Nuevo León, Monterrey, Mexico; 3 Department of Community Health Sciences, Cumming School of Medicine, University of Calgary, Calgary, AB Canada; 4 Hotchkiss Brain Institute, University of Calgary, Calgary, AB Canada; 5 O’Brien Institute for Public Health, University of Calgary, Calgary, AB Canada; 6 Clinical Research Unit, University of Calgary, Calgary, AB Canada; 7 Centre for Health Informatics, University of Calgary, Calgary, AB Canada

## Abstract

The regular use of patient-reported measures (PRMs) has been associated with greater patient satisfaction and outcomes. In this article, we will review the Calgary Comprehensive Epilepsy Program's successful experience with PRMs in both clinical and research settings, as well as our current challenges and future directions. Our experience will illustrate that is feasible and convenient to implement PRMs, and especially electronic PRMs (ePRMs), into epilepsy clinics. These PRMs have direct clinical and research applications. They inform clinical decision making through readily interpretable scales to which clinicians can expeditiously respond. Equally, they are increasingly forming an integral and central component of intervention and outcomes-based research. However, implementation studies are necessary to address knowledge gaps and facilitate adoption and dissemination of this approach. A natural symbiosis of the clinical and research realms is precision medicine. The foundations of precision-based interventions are now being set whereby we can maximize the quality of life and psychosocial functioning on an individual level. As illustrated in this article, this exciting prospect crucially depends on the routine use of ePRMs in the everyday care of people with epilepsy. Increasing ePRMs uptake will clearly be a catalyst propelling precision epilepsy from aspiration to clinical reality.

## Introduction

While clinicians have always been attentive to patients’ perspectives, it was not until the late 1970s that patient-reported outcomes (PROs) began to be applied in both clinical and research settings to formalize and optimize heath care interactions. We define PROs as outcomes directly reported by the patient, without interpretation by a third party, that pertain to that individual’s health, quality of life (QoL), or functional status (Table 1) in the context of his/her health care or treatment. We measure PROs using instruments known as patient-reported outcome measures (PROMs). In North America, the use of PROMs dates back to the Dartmouth Primary Care Cooperative (COOP) Project [1]. The regular use of PROMs has been associated with greater patient satisfaction and improved communication between health care providers and patients [2, 3]. Patient-reported experience measures (PREMs) are related to PROMs. They are tools used to capture patients’ interactions with healthcare systems and the degree to which their needs are being met, according to the York Health Economics Consortium.

**Table 1:** Definitions of selected terms related to quality of life

Term	Definition
Functional status	An individual's effective performance of or ability to perform those roles, tasks, or activities that are valued
Health-related quality of life (HR-QoL)	Physical and mental health perceptions (e.g., energy level, mood) and their correlates—including health risks and conditions, functional status, social support, and socioeconomic status
Quality of life (QoL)	Individual's perception of their position in life in the context of the culture and value systems in which they live and in relation to their goals, expectations, standards and concerns

Adapted from Higgins PT, Green S. *Cochrane Handbook for Systematic Reviews of Interventions* (Version 5.1.0); Centers for Disease Control and Prevention. *Measuring Healthy Days: Population Assessment of Health-related Quality of Life* (2000); and The WHOQOL Group. The World Health Organization Quality of Life Assessment (WHOQOL). Development and Psychometric Properties. *Soc Sci Med*. 1998;46:1569–1585

In the early 1980s, patients’ perspectives became a topic of interest in the field of epilepsy [4] in response to the growing research base of PROs in general. In the same decade, the first dedicated assessments of the QoL in people with epilepsy were reported [5]. In the 1990s, it became increasingly apparent that self-perceived health in those with epilepsy was significantly different than that of their peers, and that disease-specific evaluations would be needed to best describe their unique lived experience. The goal is to produce measures with superior responsiveness to change over time that are more capable of capturing small but clinically important changes in outcomes [6], although disease-specific and generic measures can demonstrate comparable performance in specific circumstances, such as when assessing response to antiseizure medications (ASMs) [7]. Over the next two decades, formal dimensions were defined and a plethora of epilepsy-specific (and epilepsy-targeted) PROMs were developed [8] that included the QoL in Epilepsy (QOLIE) inventories [9, 10], the Neurological Disorders Depression Inventory for Epilepsy (NDDI-E) [11], and the Epilepsy Surgery Inventory-55 [8, 12].

We have entered an era where PROMs are an essential component of outcome assessment in clinical trials. For instance, surgical resection in patients with temporal lobe epilepsy not only reduces seizure frequency, but also improves QoL [13], a decisive aspect that helps us better counsel patients who are considering temporal lobe operations during the preoperative period. However, in medically treated patients with epilepsy, reduction in seizure frequency may not be sufficient to improve health-related QoL and, therefore, seizure freedom should be the treatment target [14]. This is a critical point since reductions in seizure frequency, whilst a laudable goal given it may reduce risk of injury and death, may not have as significant an impact on QoL as complete seizure-freedom since even one seizure per year often precludes driving, confers employment restrictions on patients, and can lead to seizure-related anxiety.

## PROMs/PREMs in Alberta: the Calgary Epilepsy Program (CEP) clinical experience

The CEP maintains a patient registry on almost 6000 people that includes longitudinal PROMs/PREMs data. The CEP registry began in 2007, and we quickly realized the clinical and research potential of PROMs/PREMs. By 2009, the CEP began routinely recording measures of QoL (10-item QOLIE, ‘QOLIE-10’) [15] and depression (NDDI-E) [11], and subsequently expanded to include the EuroQoL 5-Dimension 5-Level (EQ-5D-5L) instrument, a preference-based generic health-related QoL measure [16]. These three instruments were originally selected due to their validity, reliability, low respondent burden and, in the case of the QOLIE-10, also due to its responsiveness to change.

In the CEP, seven PROMs are collected at every clinic visit and, therefore, map the longitudinal evolution of different PROs over time. Of them, two are generic PROMs: the EQ-5D-5L [16] and the 7-item Generalized Anxiety Disorder (GAD-7) scale [17] (Table 2). The remaining tools are epilepsy-specific (or epilepsy-targeted) PROMs: the QOLIE-10 [15], the Global Assessment of Severity of Epilepsy (GASE) scale [18], the Global Assessment of Disability of epilepsy (GAD) scale [19], the NDDI-E [11, 20], and the Liverpool Adverse Events Profile (LAEP) [21] (Table 3). We have not used other PROMs in the past.

**Table 2:** CEP generic PROMs, these are also available as ePROMs

PROMs	PROs	Target	Characteristics	Limitations
EQ-5D-5L	Self-perceived health state	Generic	Ranges from 1 to 100. 100 represents the best health state imaginable	High ceiling effect, low variability in effect sizes [31, 32]
GAD-7	Anxiety	Generic*	7-item scale [17]. Recommended cutoff point: > 7 [17]	

*PROMs* Patient-reported outcomes measures; *ePROMs* electronic patient-reported outcomes measures. *PROs* patient-reported outcomes; *EQ-5D-5L*: 5-level EuroQol 5-Dimension 5 instrument. *GAD-7* 7-item Generalized Anxiety Disorder scale

^*^Recently validated in people with epilepsy[17, 22]

**Table 3:** CEP epilepsy-specific/targeted PROMs, these are also available as ePROMs

PROMs	PROs	Target	Characteristics	Limitations
QOLIE-10 [15]	QoL	Epilepsy-targeted	10‐item weighted score from zero (no QoL) to one hundred (maximal QoL). Assesses seven central components, including seizure worry, emotional wellbeing, fatigue, cognition, medications, social function, and overall QoL	
GASE [18]	Epilepsy severity	Epilepsy-specific	Single‐item 0–7 scale with higher numbers representing more severe epilepsy	
GAD [19]	Epilepsy disability	Epilepsy-specific	Single‐item 0–7 scale with higher numbers representing more disabling epilepsy	
NDDI-E [11]	Depression	Epilepsy-specific	6-item scale with scores that range between 0 (no depression symptoms) to 24 (‘always or often’ answer in response to all six questions)	Unclear if able to detect change over time [24]
LAEP [21]	Side effects of ASMs	Epilepsy-specific	19 symptoms on a 4-point Likert scale [21]. The global score is achieved through simple addition; hence, scores can range from 19 (no symptoms) to 76 (‘always or often a problem’ for all symptoms)	

*PROMs* patient-reported outcomes measures; *ePROMs* electronic patient-reported outcomes measures; *PROs* patient-reported outcomes; *QOLIE-10* 10-item Quality of Life in Epilepsy Inventory; *GASE* Global Assessment of the Severity of Epilepsy; *GAD* Global Assessment of Disability; *NDDI-E* Neurological Disorders Depression Inventory for Epilepsy; *LAEP* Liverpool Adverse Events Profile

In 2019, the completion rates of PROMs/PREMs ranged from 65% (QOLIE-10) to 82% (GASE and GAD). This differential likely relates to respondent burden related to more questions within each scale. These are completion rates prior to the introduction of electronic PROMs/PREMs (ePROMs/ePREMs) that was vastly expedited in response to the COVID-19 pandemic. Our impression is that this implementation increased completion rates, especially given that to finish the form online, all questions must be answered and cannot be skipped. To facilitate uptake, especially during the pandemic, our administrative assistants routinely provide reminders to patients about the importance of completing these questionnaires over the telephone and via automated emails. We plan to formally evaluate the impact of these measures in the near future now that we are one-year into the pandemic at our clinical centre.

The GAD-7 is recommended for epilepsy care as the lifetime prevalence of anxiety disorder is higher than 20% in this patient population [22]. This scale can be used to rapidly detect generalized anxiety disorder in specialized epilepsy clinics [22]. A regular use of the NDDI-E is critical because depression occurs in up to one-third of people with epilepsy and is associated with a poor response to ASMs, more side-effects, and a higher risk of mortality [20]. The CEP uses a cut-off of > 13, which is consistent with the optimal score identified in two separate meta-analyses [20, 23]. As it is not currently clear whether the NDDI-E is able to detect change over time [24], we restrict its use to screening rather than actively treating the score itself.

We routinely use these two PROMs in clinical practice to screen and act upon mental health issues, i.e., anxiety (GAD-7) and depression (NDDI-E). This is important because the clinical interview by itself may fail to identify these conditions or to assess their severity. The NDDI-E is also useful as a screening tool about suicidality. In addition, we obtain the ‘one pager’ (see below) for every patient at every visit that, in addition to relevant clinical information, contains information about patients’ perception of the severity of their epilepsy, the disability related to seizures, their overall QoL, and their satisfaction with ASMs.

The QOLIE-10 communicates epilepsy‐specific effects on mental health and everyday functioning, including employment, driving, and social engagement [2, 15]. The GASE and GAD scales measure self-perceived epilepsy severity and disability, respectively. Higher GASE scores are associated with ongoing seizures, polypharmacy, ASM side-effects, and more severe depression and anxiety symptoms [18]. Similarly, higher scores on the GAD correlate strongly with self-perceived epilepsy severity, and moderately so with seizure frequency, current number of ASMs, ASM side-effects, and increasing severity of depression and anxiety symptoms [19]. Finally, the LAEP was developed to quantify patients’ perceptions of the side-effects of ASMs in the past 4 weeks [19, 21]. It includes 19 items, and they can be considered individually to specifically isolate symptoms related to an ASM, or globally as an overall score of tolerability.

One PREM is used alongside these PROMs (Table 4). The Treatment Satisfaction Questionnaire for Medication (TSQM) is a widely used PREM that was previously validated in a national panel study of chronic diseases [25]. In addition, this tool has been used to evaluate satisfaction with ASMs in medically [26] and surgically treated patients [25]. This patient-reported measure (PRM) is not considered a PROM as it does not assess outcomes.

**Table 4:** CEP PREM: TSQM. This is also available as ePRM

PRMs	Variable	Target	Characteristics	Limitations
TSQM	Treatment satisfaction	Generic	Primarily measures four different domains: effectiveness, side effects, convenience, and global satisfaction [26]. Higher scores indicate greater treatment satisfaction [26]	Not previously validated in people with epilepsy

*PREM* Patient-reported experience measure; *ePREM* Electronic patient-reported experience measure. *TSQM* Treatment Satisfaction Questionnaire for Medication

As PRMs are used for clinical decision making, we invite all patients to complete them before every clinical encounter. Participation is completely optional, but patients are always encouraged to participate as some of the data from the questionnaires have direct implications for the clinical encounter (e.g., depression and anxiety scores). No specific incentives are offered for completing these questionnaires. At present, patients usually complete these questionnaires using a tablet in the clinic waiting room. Administrative assistants provide patients with this tablet and the data are uploaded to a Research Electronic Data Capture (REDCap) database where they are reviewed by the clinician. We have also been experimenting with electronic data entry via emailed REDCap surveys that can be completed in advance of clinics. This process has been accelerated by the COVID-19 pandemic during which virtual clinic visits were mandated [27]. These emailed surveys are convenient and allow clinicians to prepare for clinics by reviewing ePROMS/ePREMs prior to the patient encounter. However, maintaining comparable uptake to physical encounters requires more active participation on behalf of the administrative assistants who send reminders and call patients to ensure they complete the forms. Barriers to full uptake include reliable internet access, spam filters that may automatically remove the email from the patient’s inbox, and the resources and person-power required to send reminders and explain the importance of ePROMS/ePREMs to patients. Caregivers may assist cognitively impaired patients in answering the questionnaires. In this case, the questionnaires are clearly identified as being completed by proxy, and who the proxy is.

**Fig. 1:**
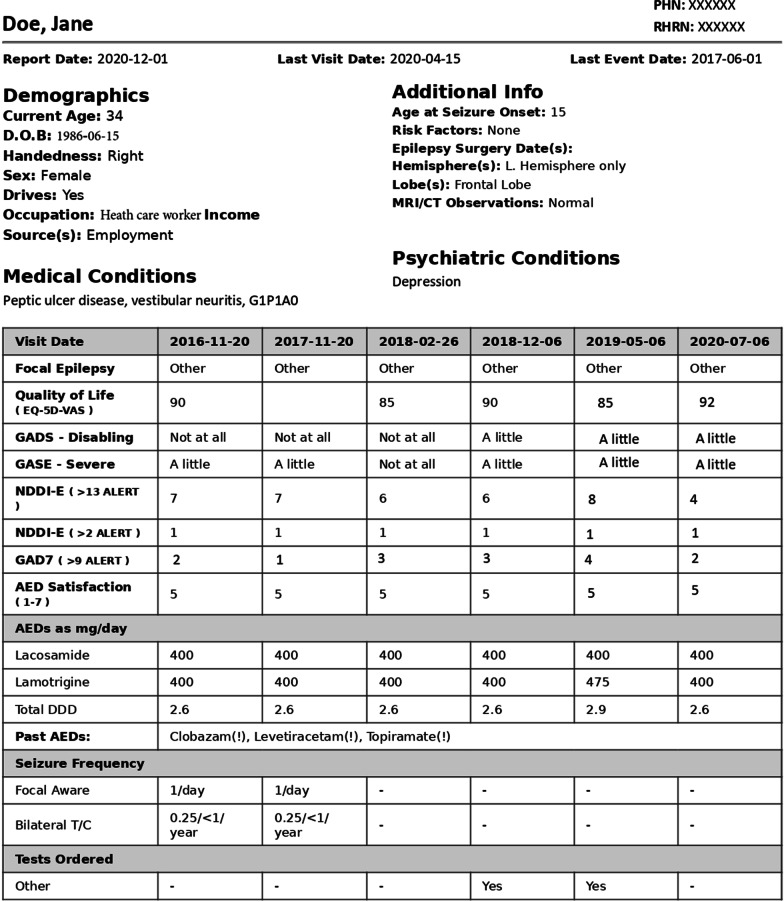
The REDCap-generated ‘one-pager,’ a PDF file summarizing relevant demographic, clinical, and PRM data. Example based on simulated data

These ePROMs/ePREMs readily inform and impact clinical practice in real time during the clinical encounter. For instance, using the REDCap tools, it is possible to automatically generate a PDF file (the ‘one pager’) summarizing relevant demographic, clinical, and PRMs data (Fig. 1). This permits a rapid and concise review of the patient’s status without having to rely on the paper or electronic chart. Another application of ePROMs is automated notification. Consultant epileptologists in the CEP are immediately alerted via email when a patient reports an NDDI-E > 13. Similar warnings can easily be deployed for a variety of outcomes including a critical rise in seizure frequency, development of ASM resistance (defined as failure of ≥ 2 appropriately chosen and dosed ASMs due to a lack of efficacy [28]), or high-risk suicidal ideation (defined as scoring > 2 on item 4 of the NDDI-E [29]). Using electronic data collection, it is possible to generate automated clinic letters immediately after the clinic visit that includes a summary of all PROMs/PREMs collected leading up to and including that day’s encounter.

We are now in the process of deploying these approaches in centres across Canada as part of the Canadian Observational Study on Epilepsy (CANOE). This initiative is intended to standardize data collection in participating centres for adult and pediatric patients with epilepsy. The overarching aim of this consortium is to study and provide new insights into the descriptive epidemiology, genetics, treatment patterns, and determinants of health outcomes of epilepsy by using the data collected during routine clinical encounters in all participating sites. All data are stored on centralized secure servers hosting REDCap with access governed by two-factor authentication. In addition to physician-led clinical inputs, PRMs form a central aspect of this data repository with longitudinal recording of all our PRMs described above. If interested in CANOE, centres across Canada can contact the corresponding author of this article.

Eight epileptologists are currently using these PROMs/PREMs in our center. In the past, three other epileptologists also used them. We show all available PROMS and PREMs on the ‘one-pager’ to facilitate clinical decision making. The intention is to provide the treating physician with immediate access to important information at the beginning of each clinical visit so issues surrounding depression (NDDI-E), suicidal ideation (item 4 of the NDD-E), and self-perceived epilepsy severity (GASE) and disability (GAD) can be readily identified and comprehensively managed, if present. Future studies will be required to gauge the degree to which access to this information truly alters clinical behaviour.

Our clinicians do not undergo formal training on PROMs/PREMs, but instructions are clearly provided to patients on the paper and electronic forms. We communicate the results of the PROMs/PREMs when evaluating and managing their medical and psychosocial health. At present, CEP PRMs are not used internally at the aggregate level to compare performance across clinicians or to evaluate interventions, though this is feasible where required.

## The CEP PROMs/PREMs and their utility in clinical research

Routine collection of ePROMs/ePREMs for clinical use also facilitates research. Patients are asked at their first clinic visit to provide consent to use these data for research purposes. The rate of consent is 88.3%, meaning that only 11.7% of people requested that their data only be used for clinical purposes. Readily available data in a digital registry permits rapid response to acutely evolving situations. For instance, from May to October 2016, there was a shortage of clobazam, a level I critical ASM, in Canada. We were able to immediately react to this situation by assessing patient views and concerns, in part using their routinely collected PROMs data. At that time, no statistically significant differences were found between the pre- and intra-shortage periods in overall and epilepsy-related QoL (QOLIE-10), fear of having a seizure over the next four weeks (QOLIE-10 item 9), and epilepsy severity (GASE) or disability (GAD) in patients who were taking clobazam. Hence, by using mixed methods approaches incorporating PROMs data, we were able to determine that patients felt supported and secure with their care despite the medication shortage [30].

In addition, we were interested in the growing use of cannabis-based treatments for epilepsy and their interaction with PROs [31]. Therefore, with readily available granular data, we explored associations between recreational cannabis use and psychosocial PROs to inform future treatment trials. Of 337 patients with epilepsy, regular cannabis use was associated with baseline depression (NDDI-E), lower QoL (QOLIE-10), and worse self-perceived epilepsy disability (GAD). Additionally, cannabis use mediated 7–12% of the effects of a premorbid psychiatric disorder on QoL and heath state valuation (EQ-5D-5L). Hence, we concluded that these data reinforce the need for uniform inclusion of PROMs in future trials evaluating the antiseizure effects of cannabidiol.

We have also begun to use PROs for promoting precision medicine through advanced analytics. After applying unsupervised machine learning techniques to five PROMs (QOLIE-10, EQ-5D-5L, NDDI-E, GASE, and GAD), we identified three discrete profiles defined by high, intermediate, and poor psychosocial health clusters [2]. Furthermore, using adjusted multinomial regression, we were able to determine that social determinants of health (requiring social assistance, inability to drive, and psychiatric comorbidities), as opposed to seizures themselves, appear to predominantly determine placement in the poor psychosocial health cluster as compared to those with high psychosocial health. This data-driven algorithm can be deployed in clinical settings via electronic health records platforms to predict the patient’s overall psychosocial health trajectory. These findings will help inform clinical decisions and interventions. Plans for these studies are underway with an initial emphasis on improving social support and careful titration of ASMs for those reporting the worst psychosocial health, in order to help optimize health for patients with epilepsy [2].

## The CEP PRMs and future directions

Epilepsy surgery is a crucial, and potentially curative option for those with drug resistant epilepsy. However, until recently, we have not had a PREM for assessing a patient’s satisfaction with surgery [25]. Therefore, we developed the Epilepsy Surgery Satisfaction Questionnaire (ESSQ-19) [25]. Patients were recruited from three centers in Canada (Calgary, Montreal, and Saskatchewan) and one in Sweden (Gothenburg). This 19-item tool has excellent internal reliability and test–retest reliability, poses minimal respondent burden, and can be used in both clinical and research settings. The ESSQ-19 has been validated in English, Canadian French, and Swedish; and Portuguese, Mandarin, and Russian validations are underway. We anticipate this can be used not only to determine *post-hoc* evaluations of epilepsy surgery satisfaction but, based on future studies, could also be used pre-operatively to counsel and advise patients regarding their likely perception of a completed operation should they choose to undergo the procedure.

In our centre, no implementation studies have been conducted to explore ways to improve PROMs/PREMs uptake by patients and to optimize application in clinical care. Such evidence on implementation will be necessary to facilitate adoption and dissemination of this approach. Ideally, through the CANOE consortium, we will be able to address this knowledge gap by running multicentre randomized controlled trials that evaluate outcomes, such as seizure-freedom, psychiatric disease severity, and treatment satisfaction, stratified by conventional care *versus* that aided by routinely and systematically collected PRM data and made available to clinical staff at each clinic visit.

## Conclusion

PROMs/PREMs are widely used in neurologic care. In general, their implementation is associated with greater patient satisfaction and improved outcomes. It is feasible and convenient to implement PROMs/PREMs, and especially ePROMs/ePREMs, into epilepsy clinics. These PRMs have direct clinical and research applications. They inform clinical decision making through readily interpretable scales to which clinicians can expeditiously respond. Equally, they are increasingly forming an integral and central component of intervention and outcomes-based research. A natural symbiosis of the clinical and research realms is precision medicine. The foundations of precision-based interventions are now being set whereby we can maximize QoL and psychosocial functioning on an individual level. As illustrated in this article, this exciting prospect crucially depends on the routine use of ePROMs/ePREMs in everyday care of people with epilepsy. Increasing ePRMs uptake will clearly be a catalyst propelling precision epilepsy from aspiration to clinical reality.

## Data Availability

Not applicable.

## Abbreviations

ASMs: Antiseizure medications
CANOE: Canadian Observational Study on Epilepsy
CEP: Calgary Epilepsy Program
COOP: Dartmouth Primary Care Cooperative
ePREMs: Electronic patient-reported experience measures
ePRMs: Electronic patient-reported measures
ePROMs: Electronic patient-reported outcome measures
EQ-5D-5L: EuroQoL 5-Dimension 5-Level instrument
ESSQ-19: Epilepsy Surgery Satisfaction Questionnaire
GAD-7: 7-Item Generalized Anxiety Disorder scale
GAD: Global Assessment of Disability of Epilepsy scale
GASE: Global Assessment of Severity of Epilepsy scale
LAEP: Liverpool Adverse Events Profile.
NDDI-E: Neurological Disorders Depression Inventory for Epilepsy
PREs: Patient-reported experiences
PRMs: Patient-reported measures
PROs: Patient-reported outcomes
PREMs: Patient-reported experience measures
PROMs: Patient-reported outcome measures
REDCap: Research Electronic Data Capture
QOL: Quality of life
QOLIE: Quality of Life in Epilepsy Inventory
QOLIE-10: 10-Item Quality of Life in Epilepsy Inventory
TSQM: Treatment Satisfaction Questionnaire for Medication

## References

[1] Wasson J, Keller A, Rubenstein L, Hays R, Nelson E, Johnson D. Benefits and obstacles of health status assessment in ambulatory settings. The clinician's point of view. The Dartmouth Primary Care COOP Project. Med Care. 1992;30(5 Suppl):MS42–9. 10.1097/00005650-199205001-00004 1583940

[2] Josephson CB, Engbers JDT, Wang M (2020). Psychosocial profiles and their predictors in epilepsy using patient-reported outcomes and machine learning. Epilepsia.

[3] Nelson EC, Eftimovska E, Lind C, Hager A, Wasson JH, Lindblad S. Patient reported outcome measures in practice. BMJ. 2015;350:g7818. 10.1136/bmj.g7818 25670183

[4] Beran RG, Read T (1980). Patient perspectives of epilepsy. Clin Exp Neurol.

[5] Soga T (1987). Restrictions on daily life's quality among people with epilepsy. Jpn J Psychiatry Neurol.

[6] Wiebe S, Guyatt G, Weaver B, Matijevic S, Sidwell C (2003). Comparative responsiveness of generic and specific quality-of-life instruments. J Clin Epidemiol.

[7] Mulhern B, Pink J, Rowen D, Borghs S, Butt T, Hughes D, Marson A, Brazier J (2017). Comparing generic and condition-specific preference-based measures in epilepsy: EQ-5D-3L and NEWQOL-6D. Value Health.

[8] Hermann BP (1995). The evolution of health-related quality of life assessment in epilepsy. Qual Life Res.

[9] Cramer JA, Perrine K, Devinsky O, Bryant-Comstock L, Meador K, Hermann B (1998). Development and cross-cultural translations of a 31-item quality of life in epilepsy inventory. Epilepsia.

[10] Devinsky O, Vickrey BG, Cramer J, Perrine K, Hermann B, Meador K, Hays RD (1995). Development of the quality of life in epilepsy inventory. Epilepsia.

[11] Gilliam FG, Barry JJ, Hermann BP, Meador KJ, Vahle V, Kanner AM (2006). Rapid detection of major depression in epilepsy: a multicentre study. Lancet Neurol.

[12] Vickrey BG, Hays RD, Graber J, Rausch R, Engel J, Brook RH (1992). A health-related quality of life instrument for patients evaluated for epilepsy surgery. Med Care.

[13] Wiebe S, Blume WT, Girvin JP, Eliasziw M. Effectiveness and efficiency of surgery for temporal lobe epilepsy study group. A randomized, controlled trial of surgery for temporal-lobe epilepsy. N Engl J Med. 2001;345(5):311–8. 10.1056/NEJM200108023450501 11484687

[14] Birbeck GL, Hays RD, Cui X, Vickrey BG (2002). Seizure reduction and quality of life improvements in people with epilepsy. Epilepsia.

[15] Cramer JA, Arrigo C, Van Hammée G, Bromfield EB (2000). Comparison between the QOLIE-31 and derived QOLIE-10 in a clinical trial of levetiracetam. Epilepsy Res.

[16] Herdman M, Gudex C, Lloyd A, Janssen M, Kind P, Parkin D, Bonsel G, Badia X (2011). Development and preliminary testing of the new five-level version of EQ-5D (EQ-5D-5L). Qual Life Res.

[17] Wang Z, Luo Z, Li S, Luo Z, Wang Z (2019). Anxiety screening tools in people with epilepsy: A systematic review of validated tools. Epilepsy Behav.

[18] Sajobi TT, Jette N, Zhang Y, Patten SB, Fiest KM, Engbers JD, Lowerison MW, Wiebe S (2015). Determinants of disease severity in adults with epilepsy: results from the neurological diseases and depression study. Epilepsy Behav.

[19] Sajobi TT, Jette N, Fiest KM, Patten SB, Engbers JD, Lowerison MW, Wiebe S (2015). Correlates of disability related to seizures in persons with epilepsy. Epilepsia.

[20] Gill SJ, Lukmanji S, Fiest KM, Patten SB, Wiebe S, Jetté N (2017). Depression screening tools in persons with epilepsy: a systematic review of validated tools. Epilepsia.

[21] Panelli RJ, Kilpatrick C, Moore SM, Matkovic Z, D'Souza WJ, O'Brien TJ. The liverpool adverse events profile: relation to AED use and mood. Epilepsia. 2007;48(3):456–63. 10.1111/j.1528-1167.2006.00956.x 17284301

[22] Micoulaud-Franchi JA, Lagarde S, Barkate G, Dufournet B, Besancon C, Trébuchon-Da Fonseca A, Gavaret M, Bartolomei F, Bonini F, McGonigal A (2016). Rapid detection of generalized anxiety disorder and major depression in epilepsy: validation of the GAD-7 as a complementary tool to the NDDI-E in a French sample. Epilepsy Behav.

[23] Kim DH, Kim YS, Yang TW, Kwon OY (2019). Optimal cutoff score of the Neurological Disorders Depression Inventory for Epilepsy (NDDI-E) for detecting major depressive disorder: a meta-analysis. Epilepsy Behav.

[24] Nixon A, Kerr C, Breheny K, Wild D (2013). Patient Reported Outcome (PRO) assessment in epilepsy: a review of epilepsy-specific PROs according to the Food and Drug Administration (FDA) regulatory requirements. Health Qual Life Outcomes.

[25] Wiebe S, Wahby S, Lawal OA, Sajobi TT, Keezer MR, Nguyen DK, Malmgren K, Tellez-Zenteno J, Atkinson MJ, Hader WJ, Josephson CB, Macrodimitris S, Patten SB, Pillay N, Sharma R, Singh S, Starreveld Y (2020). Development and validation of the Epilepsy Surgery Satisfaction Questionnaire (ESSQ-19). Epilepsia.

[26] Sweileh WM, Ihbesheh MS, Jarar IS, Taha AS, Sawalha AF, Zyoud SH, Jamous RM, Morisky DE (2011). Self-reported medication adherence and treatment satisfaction in patients with epilepsy. Epilepsy Behav.

[27] Subotic A, Pricop DF, Josephson CB, Patten SB, Smith EE, Roach P; Calgary Comprehensive Epilepsy Program Collaborators. Examining the impacts of the COVID-19 pandemic on the well-being and virtual care of patients with epilepsy. Epilepsy Behav. 2020;113:107599. 10.1016/j.yebeh.2020.107599 33238236

[28] Kwan P, Arzimanoglou A, Berg AT, Brodie MJ, Allen Hauser W, Mathern G, Moshé SL, Perucca E, Wiebe S, French J (2010). Definition of drug resistant epilepsy: consensus proposal by the ad hoc Task Force of the ILAE Commission on Therapeutic Strategies. Epilepsia.

[29] Mula M, McGonigal A, Micoulaud-Franchi JA, May TW, Labudda K, Brandt C (2016). Validation of rapid suicidality screening in epilepsy using the NDDIE. Epilepsia.

[30] Lukmanji S, Sauro KM, Josephson CB, Altura KC, Wiebe S, Jetté N (2018). A longitudinal cohort study on the impact of the clobazam shortage on patients with epilepsy. Epilepsia.

[31] Wahby S, Karnik V, Brobbey A, Wiebe S, Sajobi T, Josephson CB (2019). Cannabis use is both independently associated with and mediates worse psychosocial health in patients with epilepsy. J Neurol Neurosurg Psychiatry.

[32] Wijnen BFM, Mosweu I, Majoie MHJM, Ridsdale L, de Kinderen RJA, Evers SMAA, McCrone P (2018). A comparison of the responsiveness of EQ-5D-5L and the QOLIE-31P and mapping of QOLIE-31P to EQ-5D-5L in epilepsy. Eur J Health Econ.

